# Tinnitus perception is linked to arousal dysfunction

**DOI:** 10.1016/j.isci.2026.114729

**Published:** 2026-01-19

**Authors:** Lise Hobeika, Rémy Masson, Sophie Dupont, Alain Londero, Séverine Samson

**Affiliations:** 1 Université Paris Cité, Institut Pasteur, AP-HP, Inserm, CNRS, Fondation Pour l’Audition, Institut de l’Audition, IHU reConnect, 75012 Paris, France; 2 Sorbonne Université, Institut du Cerveau - Paris Brain Institute - ICM, Inserm, CNRS, APHP, Hôpital de la Pitié Salpêtrière, Paris, France; 3 Department of Psychology, McGill University, Montreal, QC, Canada; 4 Alan Edwards Centre for Research on Pain, McGill University, Montreal, QC, Canada; 5 Epilepsy Unit and Rehabilitation Unit, Hôpital de la Pitié-Salpêtrière, AP-HP, Centre de recherche de l'Institut du cerveau et de la moelle épinière (ICM), UMPC-UMR 7225 CNRS-UMRS 975 Inserm, Paris, France; 6 Université Paris Cité, Institut Pasteur, AP-HP, Hôpital Lariboisière, Service ORL et CCF, Unité Explorations Fonctionnelles, INSERM, Fondation Pour l’Audition, IHU reConnect, 75010 Paris, France; 7 PSITEC – Psychologie: Interactions, Temps, Emotions, Cognition, Université de Lille, ULR 4072, Lille, France; 8 Assistance Publique – Hôpitaux de Paris, GHU Pitié-Salpêtrière-Charles Foix, 75013 Paris, France

**Keywords:** Health sciences

## Abstract

Tinnitus, the perception of a phantom sound, is often associated with concentration difficulties that have not been characterized. In this study, the attentional and executive functioning of 200 participants (100 with chronic tinnitus and 100 controls) were assessed with the attentional network task, the sustained attention to response task with mind-wandering evaluations, the stroop task, and the trail making test. Tinnitus-related comorbidities (hearing loss, sleep quality, anxiety, and hyperacusis) were controlled. Individuals with tinnitus demonstrated diminished sensitivity to alerting signals and reduced sustained attention capacity, both of which are consistent with decreased levels of arousal. Unlike previously reported in the literature, we found no deficits in executive functioning specific to tinnitus, but an association was found with hearing loss and sleep disturbances. Overall, these findings support the hypothesis that tinnitus is associated with dysfunction within the arousal system, which is a new theoretical framework to study the underlying tinnitus-related cognitive complaints.

## Highlights

• Tinnitus is associated with reduced alertness and sustained attention
• Sustained attention is influenced by sleep and anxiety in individuals with tinnitus
• The findings suggest an arousal dysfunction, potentially involving the LN-CE
• Hearing loss, but not tinnitus, is associated with executive function deficits

## Graphical abstract

**Figure:**
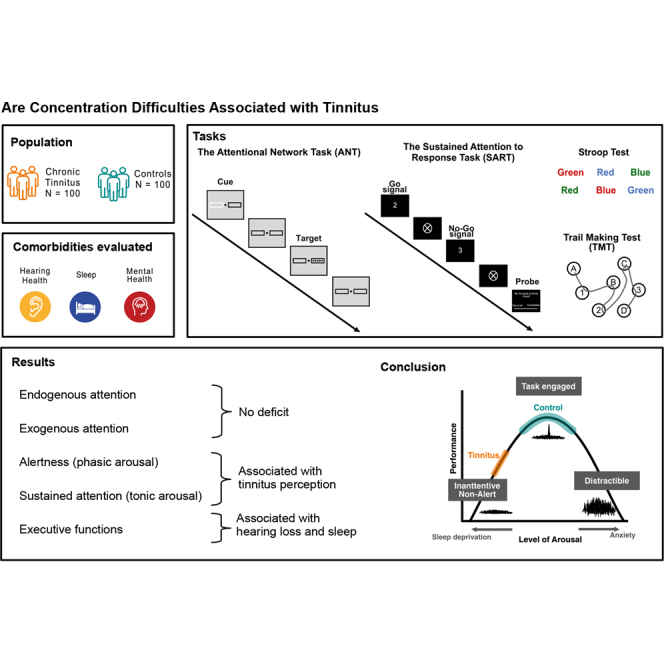

## Introduction

Continuous, non-pulsatile tinnitus, i.e., subjective tinnitus, often referred to as “ringing in the ears,” is a symptom characterized by the perception of sound in the absence of an external stimulus or internal sound source. This intriguing symptom is prevalent, affecting approximately 14% of the adult population.^1^ While tinnitus is not bothersome for most individuals, it can be highly distressing for some experiencing psychosocial disturbances such as anxiety, depression, and sleep disorders.^2^ Many individuals also report cognitive difficulties, particularly with concentration, describing an inability to filter this phantom perception out of their attentional focus.^3^ These concentration difficulties suggest possible disruptions in attentional processes, which may play a key role in the cognitive and emotional burden associated with tinnitus.^4^^,^^5^

To better understand these disruptions, it is useful to consider the attentional system as composed of multiple interconnected subsystems.^6^ Exogenous attention involves the involuntary capture of attention by unexpected or salient events, while endogenous attention refers to the voluntary allocation of cognitive resources toward goal-relevant stimuli. Both types of attention are conditioned by individuals’ arousal state, mediating alert responses (phasic arousal) and sustained attention (tonic arousal).^7^ Some theoretical models also include the contribution of executive control within the attentional system, involving inhibitory or flexibility mechanisms.^8^ In tinnitus patients, reported difficulties in concentration may reflect impairments in any of these subsystems. Consequently, a hyper-responsive exogenous attention could lead to an indiscriminate attention capture by irrelevant inputs, including the tinnitus sound. A dysfunction of the endogenous system could limit the capacity to disengage from the tinnitus percept and reorient toward relevant tasks. Alternatively, the perception of tinnitus could also disrupt arousal, inducing deficits in alertness and/or sustained attention.

The literature on tinnitus and cognition has primarily focused on attention and executive functions. Some studies report attentional impairments using divided^9^^,^^10^ or selective attention tasks,^11^ while others describe deficits in executive processes such as inhibition and cognitive flexibility.^12^^,^^13^^,^^14^ As highlighted by reviews and meta-analyses,^15^^,^^16^^,^^17^ the overall strength of the evidence remains limited because of methodological weaknesses related to the small sample sizes and the heterogeneous participant characteristics. More critically, the lack of control for comorbidities commonly associated with tinnitus severity—such as sleep deprivation, depression, and anxiety—hinders the interpretation of many studies and could lead to those mixed findings.^18^ Even hearing loss, the primary risk factor for tinnitus perception,^18^ is often inadequately controlled despite its well-documented impact on cognitive functioning.^19^ For instance, many studies report the lack of hearing impairment based on pure-tone audiometry using the conventional clinical frequency range (typically the average thresholds at 0.5, 1, 2, to 4 kHz, proposed by the World Health Organization).^20^ However, equivalent thresholds within this range do not necessarily indicate comparable hearing abilities, since hearing loss is often more pronounced at higher frequencies (6–16 kHz).^21^ As a result, the question of whether tinnitus is associated with cognitive dysfunction, particularly in the domains of attention and executive function, remains open and has never been tested within a single study.

**Figure 1:**
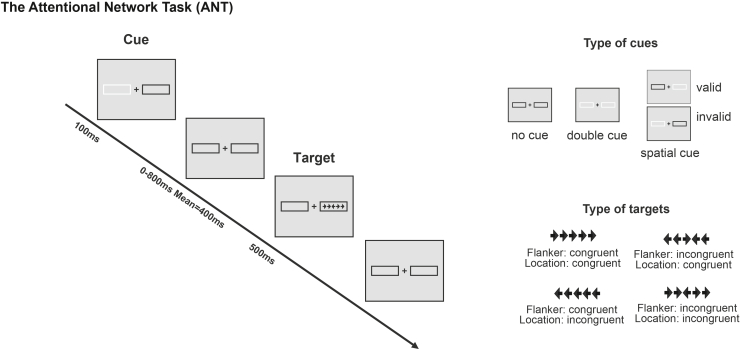
Illustration of the revised attentional network test Each trial can begin with a cue presentation, which varies depending on the type of condition (none, double, valid, or invalid). When a cue is present, a cue box flashes for 100 ms. After a variable interval (0, 400, or 800 ms), the target stimulus appears a central arrow flanked by two arrows on each side, which can be either congruent (pointing in the same direction as the target) or incongruent (pointing in the opposite direction). The target and flankers remain on screen for 500 ms. Participants respond by indicating the direction of the central arrow. Following the response, a post-target fixation period occurs, lasting between 2,000 and 12,000 ms before the next trial begins.

**Figure 2:**
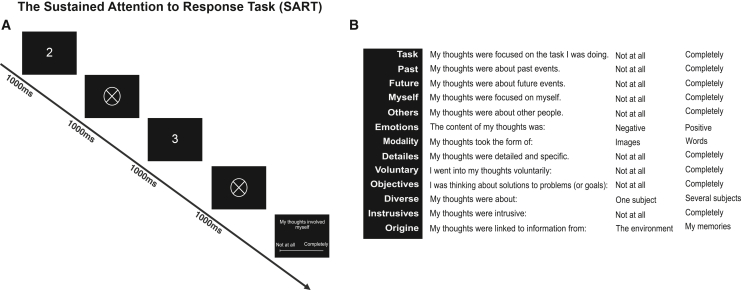
Illustration of the sustained attentional to response task (A) The task consisted of sequential digit presentations, ranging from 1 to 9. Participants were instructed to press a response button for each digit presentation, except when the no-go target “3” appeared. (B) The task was periodically interrupted by a series of 13 probe questions, randomly distributed throughout the session, which assessed the content of participants’ thoughts at the moment of interruption.

The objective of this study was to characterize both attention and executive functions in individuals with chronic tinnitus (duration >3 months) within a single study. To this end, 200 participants with and without tinnitus completed a comprehensive neuropsychological assessment including attentional and executive tasks. The attentional network task (ANT, Figure 1)^8^ was used to assess endogenous and exogenous attention, tonic and phasic arousal, as well as flexibility. The sustained attention to response task (SART, Figure 2)^22^ provided a precise measure of tonic arousal and an evaluation of the mind-wandering. The trail making test (TMT) (shifting) and the stroop task (inhibition) were used to evaluate executive functions. To minimize potential confounds related to the auditory disorder itself, only visually based tasks were administered, thereby ensuring that any observed deficits could not be attributed to the auditory modality. Moreover, we rigorously accounted for tinnitus-related comorbidities by conducting a comprehensive assessment of hearing health (audiometry with high frequencies, evoked otoacoustic emissions, distortion products otoacoustic emission, hyperacusis, as well as subjective experience of tinnitus severity for persons with tinnitus), emotional functioning (anxiety and depression), stress, and sleep deprivation. This approach, incorporating multiple measures of attention and of executive functions within a large dataset and controlling for comorbidities commonly associated with tinnitus, constitutes a distinctive strength of the study.

## Results

### Emotional and hearing comorbidities

Participants with and without tinnitus did not significantly differ in terms of age, sex ratio, laterality, and level of education (Table 1). First, we evaluated the potential group differences in emotional functioning. Tinnitus group reported significantly higher levels of anxiety, depression, perceived stress, sleep disturbance, and hyperacusis. To control for the potential confounding effects of these comorbidities, we conducted a correlation-based data reduction analysis to identify one or several scores to include in our linear model analysis of the cognitive functioning, while preventing predictors multicollinearity. Among the mental health measures, the STAI score was retained as a covariate, as it exhibited strong collinearity with all other scores (HADS-A, HADS-D, PSS4; r > 0.70, *p* < 0.001; see Table S1), rendering joint inclusion inappropriate but supporting its use as a common proxy for emotional functioning. The STAI scores showed correlations with sleep disturbances and with hyperacusis (Khalfa questionnaire), but these correlations remain below the colinearity threshold, allowing all scores to be included in the linear model (r = 0.50, *p* < 0.001, r = 0.40, *p* < 0.001, respectively). Therefore, the anxiety scores (STAI), sleep disturbances, and hyperacusis were included as covariates in the analyses of the cognitive tasks.

**Table 1:** Tinnitus and control group descriptive data

	Control	Tinnitus	Test	*p* value
	*n* = 100	*n* = 100		
**Demographic**				

Age	43 ± 13	45 ± 13	Student’s *t* test	ns
Sex (F/M)	54/46	57/43	Pearson’s chi^2^ test	ns
Laterality (left/ambidextrous/right)	13/2/85	12/0/88	Pearson’s chi^2^ test	ns
Years of education	15 ± 3	16 ± 3	Student’s *t* test	ns

**Emotional state**				

Anxiety (STAI)/80	43 ± 10	50 ± 10	Student’s *t* test	<0.001
Anxiety (HADS-A)/21	7 ± 4	10 ± 5	Student’s *t* test	<0.001
Depression (HADS-D)/21	5 ± 4	7 ± 4	Student’s *t* test	<0.001
Stress (PSS4)/16	6 ± 4	8 ± 3	Student’s *t* test	<0.001
Sleep disorder (ISI)/28	6 ± 4	8 ± 4	Student’s *t* test	<0.001

**Hearing**				

Hyperacusis (Khalfa)/42	15 ± 9	24 ± 9	Student’s *t* test	<0.001
Discomfort level (dB)/80	76 ± 10	67 ± 15	Kruskal-Wallis	<0.001
Tinnitus severity (THI)/100	–	52 ± 24	–	–
Tinnitus duration (years)	–	8 ± 10	–	–
Tinnitus central pitch (kHz)	–	6.500 ± 2.600	–	–

Demographic status, emotional state, sleep disorder, and hearing functioning of the two groups of participants. Mean scores ± SD.

Second, we measured participants’ hearing abilities with pure tone audiometry thresholds, evoked oto-acoustic emissions (EOAEs) and distortion products otoacoustic emission (DPOEA).

**Figure 3:**
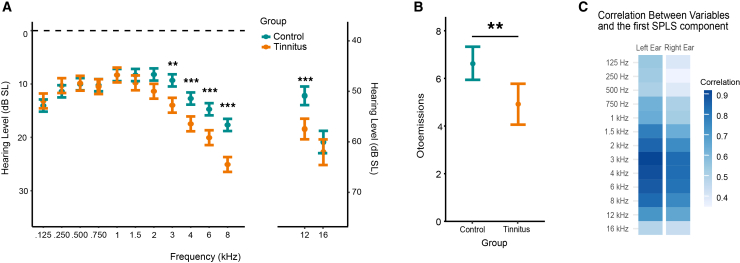
Audiological differences between tinnitus and control groups (A) Mean hearing thresholds (in dB SL) for tinnitus and control groups across frequencies ranging from 125 Hz to 16 kHz. Significant group differences were observed at frequencies from 3 to 12 kHz. (B) Mean otoacoustic emission levels showing significantly reduced responses in the tinnitus group compared to controls. (C) Derivation of a composite hearing loss score using partial least squares discriminant analysis (PLS-DA). This analysis extracted a component that optimally discriminates between control and tinnitus groups based on their hearing thresholds. Error bars represent the SEM. (∗∗ = *p* < 0.01, ∗∗∗ = *p* < 0.001, post-hoc tests with FDR correction).

*Pure tone audiometry* analysis revealed a significant effect of the factor group (Wald χ^2^ = 3.9, *p* < 0.001), of the factor frequency (Wald χ^2^ = 5,776, *p* < 0.001) as well as a significant interaction between group and frequency (Wald χ^2^ = 147, *p* < 0.001). Post-hoc tests indicated that the hearing thresholds of the control group were significantly better than those of the tinnitus group across multiple frequencies, particularly in the range of 3–16 kHz (Bonferroni post-hoc tests: *p* < 0.01 for 3 kHz, *p* < 0.001 for frequencies between 4 and 12 kHz) (see Figure 3A).

*EOAE* analysis revealed significant effect of the tested Frequency (χ^2^ [4] = 621, *p* < 0.001) with significant differences between all tested frequencies (mean values: 6.3 ± 0.8, 9.5 ± 0.8, 7.3 ± 0.8, 5.3 ± 0.8, and 0.5 ± 0.8 for the frequencies 1.189, 1.682, 2.379, 3.365, and 4.759 kHz respectively, all post-hoc <0.001 after FDR correction). There was also a significant effect of group, with higher emissions for the control group (*p* < 0.01) (see Figure 3B).

*DPOEA* analysis revealed a main effect of frequency (χ^2^ [6] = 518.2, *p* < 0.001) depicted in Figure S1 (post-hoc with FDR correction). There was no effect or interaction with the factor group.

Globally, we evidenced a deficit in the hearing abilities of participants with tinnitus compared with the controls in the high frequency range (from 3to 16 kHz). To derive a single, weighted score capturing hearing loss differences between groups, we applied a partial least squares discriminant analysis (PLSs-DA) on the auditory thresholds of the left and right ears from 125 to 16 kHz. This supervised multivariate method reduces dimensionality while assigning greater weight to the auditory thresholds most discriminative between groups. The first component explained 46% of the variance; the correlations between the component and the original variables are represented in Figure 3C. As expected, the higher frequencies (above 2 kHz) were more correlated with the component than the lower frequencies (below 2 kHz), confirming that the hearing deficits between the groups are larger in higher pitches. We used the first component as a hearing loss score to control for the effect of this comorbidity in the linear models.

### Dysfunction of the arousal system associated with tinnitus

We used two tasks (ANT and SART) to assess the attentional abilities of participants with and without tinnitus. All analysis included the emotional and hearing scores, as reported in the preceding steps, to account for the potential effects of those tinnitus comorbidities on cognitive functioning.

### Attentional network task

The analysis focused on the mean RTs, calculated after excluding error trials (incorrect and missing responses), since the mean errors was very low (3.6% ± 0.03). The analyses of the *endogenous orientation*, *exogenous orientation*, *and Flanker conflicts* revealed a deceleration with age (Wald χ^2^ = 21.6, *p* < 0.001), and significant effects of the cues (Wald χ^2^ = 195 - 1,670, *p* < 0.001), confirming that the experimental manipulations worked. Participants were faster with a valid cue (801 ± 9 ms) than a double cue (845 ± 9 ms), with a valid cue (795 ± 9 ms) than after an invalid cue (882 ± 9 ms), and with a congruent cue (748 ± 8 ms) than an incongruent cue (913 ± 8 ms). There were significant main group effects (Wald χ^2^ = 4.9–5.9, *p* < 0.05), the reaction times of participants with tinnitus were globally slower than those of controls. There was no significant interaction between the cues and the group, indicating no evidence of dysfunction in these attentional subsystems associated with tinnitus.

**Figure 4:**
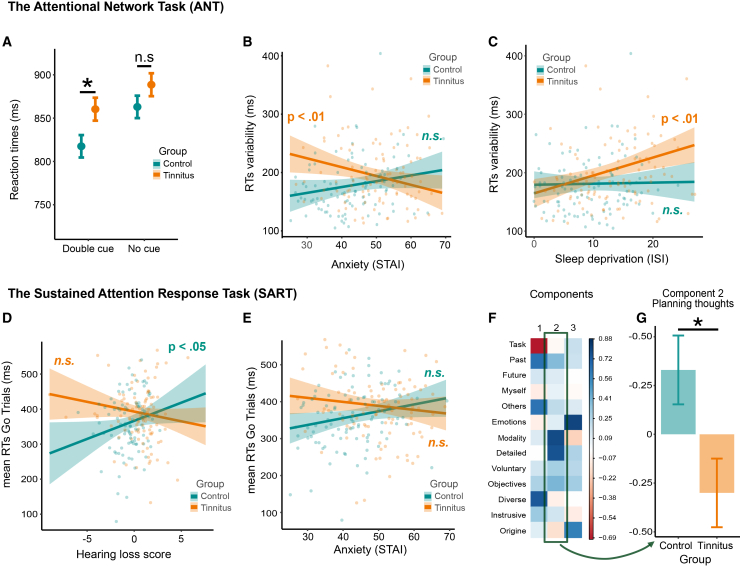
Attentional differences between tinnitus and control groups (A–C) ANT results (A). Tinnitus group showed a reduced alerting effect compared to control, characterized by less sensitivity to double cues compared to a situation without cue. (B and C) RTs variability, a measure of sustained attention, was higher in the tinnitus group. There was an interaction with both anxiety (B) and the sleep deprivation (C). Specifically, in tinnitus participants, sustained attention improved with increased anxiety and deteriorated with sleep deprivation, whereas control participants maintained stable performance across these variables. (D–G) Results of the SART. (D and E) Mean reaction times during the SART were significantly higher in the tinnitus group, indicating impaired sustained attention. Significant interactions were observed with hearing loss (D) and anxiety (E), demonstrating that these factors differently modulate of sustained attention between groups. (F) Principal-component analysis on thought content during SART task interruptions revealed three components: task-related thoughts (component 1), planning-related thoughts (component 2), and memory-related thoughts (component 3). (G) Between-group analysis of component 2 revealed significantly more planning-related thoughts in the control group compared to the tinnitus group. Error bars represent the SEM, error ribbons represent the 95% CI. (∗ = *p* < 0.05, ∗∗∗ = *p* < 0.001, in A: post-hoc tests with FDR correction, in G: result of a linear mixed model).

Finally, we analyzed the functioning of the arousal system: the alert and sustained attention. Analysis of the *alerting* revealed a deceleration with age (Wald χ^2^ = 21.6, *p* < 0.001), significant effects of the type of cue (Wald χ^2^ = 113.5, *p* < 0.001), of the group (Wald χ^2^ = 4.6, *p* = 0.03) and a significant interaction between group × type of cue (Wald χ^2^ = 4.7, *p* = 0.03). As seen in Figure 4A, Tinnitus participants were slower than Control participants after a double cue (planned comparison: *p* = 0.01, Bonferroni correction), but there was no group difference in the absence of a cue. This result indicates a lower speeding benefit of an alerting cue in presence of tinnitus.

The analysis of the *Sustained attention* revealed an effect of the group (F [1,188] = 3.8, *p* = 0.05) with more variable RTs in the tinnitus group. Analysis revealed an interaction between group × anxiety (F [1,188] = 7.9, *p* = 0.005): the RTs variability of participants with tinnitus decreased as anxiety increased (slope different from zero: *p* =0 .02), which was not the case for control participants (Figure 4B).There was also an effect of sleep disorder (F [1,188] = 6.3, *p* = 0.01), as well as an interaction between group × sleep disorder (F [1,188] = 4.6, *p* = 0.03): the RTs variability among participants with tinnitus increased with sleep disorders (slope different from zero: *p* =0 .001), which was not the case for control participants (Figure 4C). Globally, participants with tinnitus presented a deficit in sustained attention, modulated by their level of anxiety and sleep, which was not the case for control participants.

### Sustained attention to response task

We used the SART to study more closely the sustained attention abilities and evaluate mind wandering in this task thanks to series of probes.

*Sustained attention* is linked in this task to no go trials accuracy and go trials RTs. The analysis of the *no go trial’s accuracy* revealed an effect of age (F [1,185] = 8.3, *p* = 0.005) and sleep (F [1,185] = 5.8, *p* = 0.02), accuracy on no go trials being higher with aging and lower with sleep deprivation. There was no effect of the group on this measure. Conversely, analysis of the *RTs go trials* evidenced a deceleration with age (Wald χ^2^ = 13.7, *p* < 0.001), effects of the group (F [1,185] = 4.3, *p* = 0.04) with slower RTs in the tinnitus group, an interaction between group and anxiety (F [1,185] = 4.7, *p* = 0.03) with RTs decreasing with anxiety in the tinnitus groups and increasing in the control group (slopes are not statistically different from 0) (Figure 4D). Analysis also revealed an interaction between group and hearing loss (F [1,185] = 6.4, *p* = 0.01), with higher RTs being associated with hearing loss in the control group only (slope *p* <0 .05), but not in the tinnitus group. (Figure 4E).

The answers to the *mind wandering* probe questions were first analyzed using a principal-component analysis, with the extraction of three components (see Figure 4F). According to the highest loadings associated with each component, we can describe the first component as thoughts related to the task, to events from the past, thoughts on others and several subjects. The second component was thoughts using words, specific, on solution or objectives. The third was thoughts with an emotional valence, from information in their memory.

The analysis of the *first component* (task-related thoughts) *and third component* (emotional thoughts) did not reveal effects of the group (see full analysis in supplemental information, section S1). In opposition, the analysis of the *second component* (planning thoughts) indicated an effect of the level of education (Wald χ^2^ = 5.0, *p* = 0.02), with more planning-related thoughts being related to a higher education level. There was an effect of the group (Wald χ^2^ = 5.4, *p* = 0.01), indicating that participants with higher education exhibited more planning-related thoughts, while the control group demonstrated significantly more planning thoughts than the tinnitus group (see Figure 4G).

### Robustness of results on arousal: Sensitivity analysis

To verify the robustness of the current findings, we ran the analysis on a subgroup of the participants to ensure a perfect match in terms of hearing abilities, sleep disorder and emotional functioning between the control (*n* = 47) and the tinnitus (*n* = 47) groups (see Table S2; Figures S2A–S2C). The results of these new analyses confirmed (i) the effect of group on alerting (Figure S3A), (ii) the interactions between group and anxiety, and group and sleep deprivation on sustained attention in the ANT (Figures S3B and S3C). In the analysis of the SART RTs, we evidenced a marginal interaction between group and anxiety (Figure S3D). In the mind wandering task, the effect of group on planning thoughts was replicated (Figures S3E and S3F). Overall, these supplementary analyses ran on a sub-group of well-matched participants confirmed the robustness of the findings on the arousal dysfunction associated with tinnitus (see full analyses in supplemental information, section S2).

### Associations between sustained attention abilities and tinnitus severity

We tested if the scores that differ between groups (ANT alerting effect, ANT RTs variability, and SART mean RTs) were linked to tinnitus characteristics. We used as cognitive scores the partial scores computed by the linear models described above, to correct for the possible influence of the confounding factors (age, sex, level of education, hearing loss, anxiety, sleep disorder, and hyperacusis). The alerting effect was computed individually by subtracting the RTs in the no cue condition from the double cue condition. The subjective experience of tinnitus severity was evaluated with the THI questionnaire (global THI score) and with the THI functional subscale that refers to the interaction of tinnitus with daily life, including concentration, sleep, or hearing. We also tested the relationship between the cognitive scores and tinnitus duration (in years).

We evidenced a correlation between ANT RTs variability and THI functional score (r [98] = 0.26, *p* =0 .04, FDR correction). Analysis also revealed a correlation between the SART RTs and the THI scores (r [98] = −0.30, *p* =0.01, FDR correction), and its functional subscale (r [98] = −0.25, *p* = 0.03, FDR correction), (Figure S4). There were no other significant correlations.

### Executive functions deficit is associated with hearing loss

Finally, we studied the executive functioning in presence of tinnitus, using the TMT and stroop tests. Analyses of the *TMT* and the *stroop* revealed effect of hearing loss (F [1,186] = 4.8–6.1, *p* = 0.01 - 0.03) with better performances associated with lower hearing loss. Analyses also revealed an effect of sleep on the stroop test (F [1,186] = 6.7, *p* = 0.01), with worst performances associated with sleep deprivation. There was also an effect of the level of education on the TMT scores (F [1,186] = 4.7, *p* = 0.03). There was no effect of the factor group.

## Discussion

In this study, we investigated the attentional functions (both endogenous and exogenous orientation), the arousal (alert and sustained attention), and the executive functions associated with tinnitus in a large sample of participants with tinnitus matched to control participants in terms of age, sex, and education level. As the tinnitus and control groups differed in hearing abilities, emotional state, and sleep deprivation, these factors were included as covariates in the linear models to account for their potential influence. In participants with tinnitus, we evidenced a deficit in phasic alertness, i.e., the speeding following the perception of a salient event and a deficit in sustained attention in relation with anxiety, sleep, and hearing loss. We confirmed the robustness of those findings with a sensitivity analysis that consists of the creation of two subgroups matched on these comorbid factors. Unlike the data reported in the literature, we did not find a link between executive functions and tinnitus perception. However, we did find an effect with tinnitus-associated comorbidities, namely hearing loss and sleep deprivation, indicating that executive functions deficit may be more closely related to these associated conditions than to tinnitus itself.

Before addressing the main findings, it is important to consider the auditory and emotional profiles of participants with tinnitus in this study. Consistent with previous literature, individuals with tinnitus exhibited higher levels of stress, anxiety, depression, and sleep disturbances compared to controls.^16^ Despite pure-tone audiometry suggesting no hearing impairment in the tinnitus group,^20^ they showed subtle but consistent deficits in hearing function, including elevated thresholds and reduced distortion product amplitudes. Otoacoustic emissions remained comparable across groups. In the analysis of attention and executive tasks, we carefully accounted for tinnitus-related comorbidities such as emotional, sleep-related, and auditory disturbances. Since hearing loss in our sample predominantly affected high frequencies (at 4 kHz and above), we avoided using the conventional clinical frequency range (typically, the average thresholds at 0.5, 1, 2, and 4 kHz, proposed by the World Health Organization^20^), we used a PLSs approach to capture group differences more accurately. To further isolate the specific contribution of tinnitus, we included hyperacusis, a common comorbidity affecting approximately 80% of individuals with tinnitus^23^ as a covariate. This comprehensive approach ensured that our conclusions were linked to the tinnitus itself, rather than its associated hearing and emotional conditions.

Group differences in attentional functioning were observable for alertness and sustained attention. Specifically, participants with tinnitus experienced reduced benefits from alerting cues, suggesting either a lower bump of phasic arousal following the warning cue or a diminished impact of this bump on performance. Moreover, participants with tinnitus exhibited slower reaction time in the SART and greater reaction time variability in the ANT, both of which are indicative of diminished sustained attention.^24^^,^^25^ These results suggest that tinnitus negatively impacts tonic alertness, maybe reflecting a poorer internal ability to modulate arousal to an optimal level for task performance. Interestingly, sleep deprivation and anxiety effects on sustained attention were significantly more pronounced in the tinnitus group than in the control group. This finding suggests that individuals with tinnitus are less able than controls to compensate or to cope with the adverse effects of sleep deprivation. Furthermore, unlike control participants, anxiety modulates sustained attention in individuals with tinnitus, improving their performance. Finally, the link between sustained attention difficulties and subjective experience of tinnitus severity, notably cognitive functioning complaints, strengths the idea that concentration difficulties commonly reported by individuals with tinnitus may be related to sustained attention deficits.

Patterns of mind-wandering also differed between groups. Control participants reported more frequent problem-solving thoughts during the task, while those with tinnitus were less likely to engage in such task-unrelated thoughts. It might be possible to interpret this result by differences in metacognitive awareness, reporting biases, or motivation levels between participants with tinnitus and controls. However, if these factors were responsible for this pattern of mind-wondering, we would expect to observe group differences across the three types of thoughts. Since it was not the case, we suggest that individuals with tinnitus may have had fewer cognitive resources at their disposal, making it more difficult to shift attention away from the task compared to control participants. This is also in line with a potential disruption of tonic arousal in tinnitus, as arousal mediates to amount of available cognitive capacity during a task.^26^

Importantly, all cognitive tasks involved visual stimuli only, to avoid any potential interference from the auditory disorder itself. As the phasic and tonic arousal systems are thought to be largely supramodal,^27^^,^^28^ our results reflect general-domain attentional dysfunction. According to the literature, phasic alertness and tonic sustained attention are both underpinned by a common neurobiological mechanism: the locus coeruleus-norepinephrine (LC-NE) system.^7^^,^^29^ This subcortical system critically regulates cortical excitability and sensory processing through distinct activity patterns. We can, therefore, propose that brief phasic bursts triggered by salient events generate momentary alerting responses, while sustained tonic activity maintains general arousal and vigilance, thereby supporting sustained attention. It has been suggested that LC-NE effects on cognition follows an inverted-U curve.^30^ Moderate LC-NE activity enhances attention to an optimal level, whereas low activity (e.g., due to fatigue or drowsiness) can lead to mind-wandering and attentional lapses. Excessive levels (e.g., under stress) produce hypervigilance, and distractibility. Our study revealed that participants with tinnitus exhibited reduced tonic sustained attention, interacting with sleep deprivation and anxiety, and decreased phasic alertness. These findings suggest that the LC-NE system could be a good candidate to understand tinnitus related attention deficits. A chronic hypoactivity within this critical neuro-modulatory system could explain why sleep deprivation and anxiety exhibit compounded effects in tinnitus compared to the general population. However, additional studies involving direct measures of the LC-NE activity are needed to confirm this hypothesis.

Executive function deficits have been repeatedly reported in individuals with tinnitus.^13^^,^^14^ However, the evidential strength of these findings remains limited, largely due to inadequate control of tinnitus-related comorbidities and small sample sizes.^15^^,^^16^^,^^17^ Contrary to previous reports, we found no direct link between tinnitus and executive dysfunction; rather, deficits in executive functions, and in particular in cognitive inhibition and flexibility, were attributed in the current study to hearing loss and sleep deprivation. This is consistent with studies showing associations between hearing loss and executive function both in the general population^31^^,^^32^ and in tinnitus samples.^33^ Our findings suggest that executive deficits previously reported in persons with tinnitus may reflect the impact of tinnitus-related comorbidities rather than tinnitus itself. The results of the current study underscore the importance of rigorously controlling possible confounds related to comorbidities in studies aiming at characterizing the cognitive difficulties associated to tinnitus.

In this study, we identified an association between tinnitus perception and dysfunctions within the arousal system, interacting with sleep and anxiety factors. An important remaining question concerns the causal relationship among these symptoms. The prevailing hypothesis suggests that tinnitus disrupts attentional processes by persistently capturing attention due to its high salience, which in turn may lead to sleep disturbances and heightened anxiety.^16^^,^^34^ Conversely, pre-existing attentional dysfunction may contribute to the development of chronic tinnitus.^35^ Sedley and collaborators have proposed that attention difficulties can lead to an abnormal focus on the tinnitus percept, thereby increasing its salience, altering perceptual priors, and facilitating chronicity.^36^ A third possibility is that tinnitus perception and the dysregulation of attention, sleep and anxiety share a common underlying mechanism. Our results implicate the arousal system in the pathophysiology of tinnitus, a system intricately linked to attention, sleep regulation, and anxiety. We hypothesize that dysregulation within this system could concurrently precipitate all these interrelated disturbances. Further studies, particularly longitudinal designs, are warranted to elucidate the directionality and causal relationships among tinnitus, attention, sleep, and anxiety.

By rigorously controlling for key comorbidities, namely hearing loss, emotional distress, and sleep disturbances thanks to a large sample size and a sensitivity analysis, our study succeeded in disentangling the cognitive deficits genuinely associated with tinnitus from those attributable to its frequent comorbid conditions. We propose a novel theoretical framework for future studies in which dysregulation of arousal may mediate both tinnitus perception and its interactions with sleep and anxiety. This integrated model opens new avenues for understanding and targeting the cognitive and emotional burden of tinnitus, that deserve to be further investigated with longitudinal or interventional studies, to determine whether arousal dysfunction precedes or results from chronic tinnitus.

### Limitations of the study

The conclusion of the study, namely, that tinnitus is associated with arousal dysfunction, possibly supported by the LC-NE system, is based on behavioral measures. Further research should focus on direct measurements of the arousal system and LC-NE activity to confirm these conclusions. Moreover, this cross-sectional study evidences an association between tinnitus and arousal but cannot establish causality. Longitudinal studies are necessary to address this question.

## Resource availability

### Lead contact

Further information and requests for resources and information should be directed to and will be fulfilled by the lead contact, Lise Hobeika (lise.hobeika@gmail.com).

### Materials availability

This study did not generate new unique reagents.

### Data and code availability

• Data are available at https://zenodo.org/records/18048030.^37^
• The R markdown codes written to perform the statistical analysis are available on Gitlab (https://gitlab.pasteur.fr/lhobeika/tinnitus-studies/-/tree/main/Arousal%20study).
• Any additional information required to reanalyze the data reported in this article is available from the lead contact upon request.

## Acknowledgments

The project was supported by the 10.13039/100019671Fondation Pour l’Audition (FPA RD-2019-10). This work has benefited from a French government grant managed by the 10.13039/501100001665Agence Nationale de la Recherche under the France 2030 program, reference ANR-23-IAHU-0003. L.H. was funded by the European Union’s 10.13039/100018693Horizon Europe Framework Program (HORIZON) under the Marie Skłodowska-Curie Postdoctoral Fellowship (grant No. 101146406) and the Fondation des Gueules Cassées.

## Author contributions

All authors conceived and designed the study. L.H. collected the data, performed the statistical analyses, and drafted the manuscript. All authors were involved in the interpretation of data, and critical revision of the manuscript.

## Declaration of interests

Authors declare no conflict of interest.

## STAR★Methods

### Key resources table

**Table:** 

REAGENT or RESOURCE	SOURCE	IDENTIFIER
**Deposited data**		

Data	Zenodo	https://doi.org/10.5281/zenodo.18048030

**Software and algorithms**		

MedRx AVANT	MedRx	https://medrx-diagnostics.com/products/software
OAE-Suite software	Interacoustics	https://www.interacoustics.com/abr-equipment/eclipse
MATLAB 2020a	MathWorks	https://mathworks.com
Psychtoolbox-3	Brainard^38^	http://psychtoolbox.org
R version 4.2.2	R Core Team	https://www.r-project.org/
R Studio version 2025.09.0 + 387	Posit Team 2025	https://posit.co/download/rstudio-desktop/
Analysis codes	Deposited on Gitlab	https://gitlab.pasteur.fr/lhobeika/tinnitus-studies/-/tree/main/Arousal%20study

### Experimental model and study participant details

#### Participants

Two hundred participants recruited at the Hôpital Européen Georges Pompidou (HEGP, Paris) were divided in two groups: a Tinnitus group (n = 100), with individuals who have experienced subjective (non-pulsatile) chronic tinnitus for more than three months, and a Control group (n = 100), comprising individuals without tinnitus matched to the tinnitus group in terms of age, sex and education level. Gender identity was not collected. Inclusion criteria were as follows: age between 18 and 65 years, absence of severe or profound hearing loss (no unilateral mean hearing loss exceeding 65 dB at 0.5 kHz, 1 kHz, 2 kHz, and 4 kHz^20^), normal or corrected-to-normal vision, absence of neurological disorders, and fluency in French. The study was approved by an institutional review board (CPP Sud-Est VI, Clermont-Ferrand, France; reference: AU 1643) and registered on ClinicalTrials.gov (first posted on 15/01/2021, identifier NCT04717388). All procedures were conducted in accordance with relevant guidelines and regulations. Informed written consent was obtained from all participants prior to participation.

### Method details

#### Socio-emotional and demographics

Information about participants’ demographic (age, sex, educational level), mental health with the Handicap Anxiety and Depression State (HADS),^39^ the Spielberger Trait Anxiety Inventory (STAI-Y B),^40^ the Perceived Stress scale (PSS-4)^41^ and sleep quality with the Insomnia Severity Scale (ISI)^42^ were collected. The ancestry and race of participants were not recorded, as this information is considered sensitive data under French law and was not covered by the current ethics approval.

#### Auditory and tinnitus evaluations

All participants underwent an auditory evaluation, including a pure tone audiometry testing frequency from 125Hz, 250Hz, 500Hz, 750Hz, 1kHz, 1.5kHz, 2kHz, 3kHz, 4kHz, 6kHz, 8kHz, 12kHz and 16kHz using the MedRx AVANT software. Auditory thresholds at the high frequencies (12kHz and 16kHz) were measured in dB HL and converted in SL using norms.^43^ Participants who did not perceive them were assogned the maximum value. Participants also performed oto-emissions at 1.189 kHz, 1.682 kHz, 2.379 kHz, 3.365 kHz, 4.759 kHz, and distortion products at 1.230 kHz, 1.641 kHz, 2.461 kHz, 3.281 kHz, 4.915 kHz, 6.555 kHz, 8.196 kHz. Measures were collected using the Interacoustics Eclipse with the OAE-Suite software.

Hyperacusis was evaluated using the Khalfa questionnaire^44^ and with a measure of the discomfort level at 1kHz (with a maximum at 80dB) separately for each ear, then average to get one score. Patients with tinnitus answered questions about the tinnitus characteristics (i.e. time since onset, localization, type of sound…) taken from the ESIT-SQ,^45^ filled the Tinnitus Handicap Inventory questionnaire (THI), a questionnaire evaluating the subjective experience of tinnitus severity, including a subscale about cognitive functioning.^46^ Additionally, they performed a tinnitus matching that allows to create a sound matching the tinnitus percept by choosing the loudness, the central pitch, a type of noise around this central pitch and a rhythm, using the MedRx tinnometer.

#### Attentional Network task (ANT)

We used the revised Attentional Network task (ANT-R),^8^ coded with Matlab 2020a and the Psychtoolbox-3.^38^ The task presents stimuli consisting of five horizontal black arrows in a row (one central arrow with four flankers) pointing leftward or rightward. The task is to identify as quickly and accurately as possible the central arrow’s direction by pressing a key with the index finger (left hand for a leftward direction, right hand for a rightward direction). Reaction times (RTs) were collected up to 1700ms after target onset.

A cue in the form of a 100ms flashing box could precede the target (by 0, 400, or 800ms) under three conditions: i) No-cue: no flashing cue box. ii) Double-cue: both boxes flash, providing temporal information only, iii) Spatial-cue: one box flashes, offering both temporal and spatial information. Intertrial intervals ranged from 2000-12000ms (mean=4000ms). A fixation cross was visible at the center of the screen throughout the duration of the task. The protocol is illustrated in Figure 1.

The experimental conditions include the factors: Cue (no-cue, double-cue, invalid spatial-cue, valid spatial-cue, with three times more presentation of valid-spatial cue than the other combinations), Target location (left, right), Target directions (left, right), Flanker congruencies (congruent, incongruent) and the Cue-to-target delays (0, 400, and 800ms). The combination of those conditions leads to 144 trials, split into 2 runs of 72 counterbalanced trials. Based on those conditions, we defined different conditions of interest. The valid/invalid trials were trials with spatial cues (left/right), with a similar/different spatial position than the target (left/right). The congruent/incongruent trials were trials with similar/different orientation of the target arrow (left/right) and the flanker arrows (left/right). Different scores were computed based on the different conditions to assess the function of each attentional subcomponents.

• **Endogenous orientation:** Reaction time benefit of a lateralized valid cue, comparing *RTs double cue with RTs valid cue*.
• **Exogenous orientation:** Reaction time benefit of a valid cue compared to an invalid one, comparing *RTs invalid cue with RTs valid cue*.
• **Alerting:** Reaction time benefit of an alerting cue comparing *RTs no cue with RTs double cue*.
• **Sustained Attention:** Variability in reaction times, calculated as *standard deviation of RTs* throughout the task.
• **Executive Control (Flanker Conflict Effect):** Reaction time cost of inhibiting the distracting incongruent flankers, comparing *RTs flanker incongruent with RTs flanker congruent*

#### Sustained attention to response task (SART)

We used the ordered SART (Figure 2A), coded with Matlab 2020a and the Psychtoolbox-3.^38^ This is Go/No-Go paradigm is composed of sequential presentation of numbers from 1 to 9 were presented sequentially, in ascending order (not randomly). Participants had to press the ‘alt’ key of the keyboard whenever a number appears, except when the number is three, in which case they must withhold their response. Each number remained on the screen for one second, followed by a one-second display of a circle. Each number was presented 33 times throughout the task. Periodically, the task paused, and participants responded to a series of 13 questions adapted from^47^ assessing mind wandering (Figure 2B). Participants had to rate on a 1 to 10 scale whether their thoughts were: *on the task, about past events, about future events, about themselves, about others, negative or positive, in form of images or words, detailed and specific, about solutions to problems, about one or several subjects, intrusive, linked to information from the environment or their memory,* and finally *if they went into their thoughts voluntarily*. There were 10 sets of questions. We calculated the accuracy on the no-go trials, the mean RTs as indicators of participants sustained attention. Answers to the probe questions were analyzed with a principal component analysis, from which we extracted three components in line with.^48^

#### The Stroop and Trail making test (TMT)

We used the classical Stroop color-word test to evaluate inhibition.^49^ The task consists of three main conditions: 1. Color condition: identify the color of rectangles. 2. Reading condition: reading color words printed in black ink. 3. Interference Condition: naming the color of the ink of color words while inhibiting the reading of the word (e.g., if the word “RED” is written in blue ink, the answer is “blue”). Participants were instructed to be as quick and accurate as possible. The time difference between the reading and interference conditions represented the inhibition ability.

The TMT is composed of two conditions.^50^ In condition A, participants had to connect numbers in ascending order as fast as possible (1, 2, 3…). This condition aimed to measure visuo-motor processing speed. In condition B, participants had to alternate between numbers and letters in ascending and alphabetical order, respectively (1, A, 2, B…), which additionnally requires cognitive flexibility. We analyzed the time difference between the two conditions as a measure of cognitive flexibility ability. Participants were instructed to do the task as quickly and accurately as possible.

### Quantification and statistical analysis

All analysis were run using the software R version 4.2.2 with R Studio version 2025.09.0+387. The hearing thresholds, oto-emissions and distortion products were analyzed using linear mixed-effects model including as fixed effects the factors Age, Sex (Female/Male), Ear (Left/Right), Group (Tinnitus/Control), Frequency, the interactions between the factors Frequency × Group, Age × Group, and the factor Subject as random effect.

Data were analyzed using a linear mixed-effects model (LMM), including as fixed effects the factors Age, Sex, Education level, Group, Hearing loss, Hyperacusis, Sleep disorder, Anxiety, Cue and the interactions between the factors Cue x Group. The factor Subject was included as a random factor. We applied a logarithmic transformation on the means RTs to meet the LMM assumptions (normality of the residuals, homoscedasticity).

All analysis except the sustained attention included a two levels factor cue, with levels for each attentional subfunction: valid/invalid for Exogenous orientation, valid/no cue for Endogenous orientation, no cue/double cue for the Alerting and Conflict/no conflict for the Executive.

In the SART, the TMT and Stroop, all data were analyzed using linear model with the factors Age, Sex, Education level, Group, Hearing loss, Hyperacusis, Sleep disorder, Anxiety, and the interactions between the Group and the factors Hearing loss, Sleep disorder and Anxiety We applied a logarithmic transformation on the means RTs to meet the model assumptions (normality of the residuals, homoscedasticity).

Sex was included as a covariate in all statistical analyses to account for its potential influence on cognition; no sex-specific effects were observed unless explicitly reported. In the figures, significance values are marked with asterisks, indicating ∗ p < .05, ∗∗ p < .01 and ∗∗∗ p < .001.
