# Supplementary material for: Tinnitus perception is linked to arousal dysfunction
Source: iScience. 2026 Jan 19;29(2):114729. doi: 10.1016/j.isci.2026.114729 (PMC12907670; doi:10.1016/j.isci.2026.114729)
Supplement: Document S1. Figures S1–S4, Tables S1 and S2, and Data S1 [file mmc1.pdf]

iScience, Volume *29*

Supplemental information

Tinnitus perception is linked to arousal dysfunction

Lise Hobeika, Rémy Masson, Sophie Dupont, Alain Londero, and Séverine Samson

Figure S1: Distortion products Otoacoustic Emissions (DPOEA) level of each tested frequency. Error bars represent the SEM.

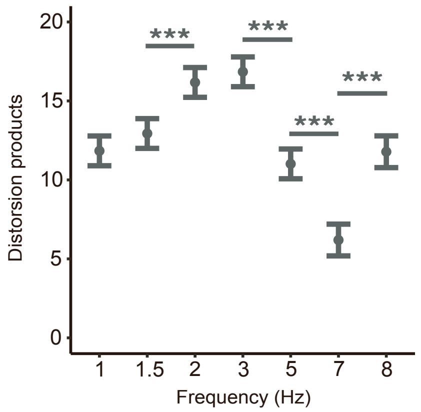

Figure S2: **Matching of hearing between the tinnitus and control subgroups. A.** Mean hearing thresholds (in dB SL) for tinnitus and control groups across frequencies ranging from 125Hz to 16kHz. **B.** Mean Evoked Otoacoustic Emissions (EOAE) levels **C**. Mean Distortion products Otoacoustic Emissions (DPOEA) levels. All measures show no evidence of group differences. Error bars represent the SEM.

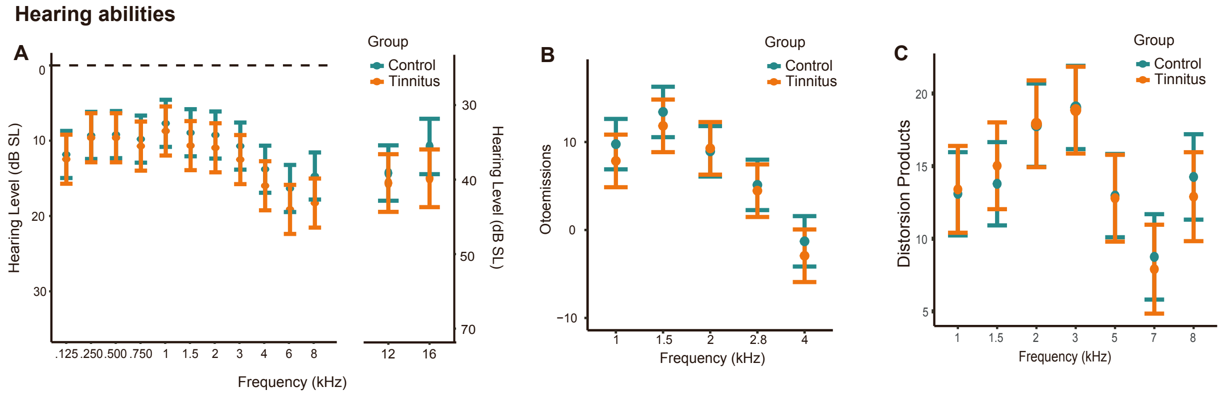

Figure S3: Attentional differences between tinnitus and control subgroups. A to C: ANT Results A. Tinnitus group showed a reduced alerting effect compared to control, characterized by less sensitivity to double cues compared to a condition without cue. **B and C.** RTs variability, a measure of sustained attention, was higher in the tinnitus group. There was an interaction with both anxiety (B) and the sleep deprivation (C). Specifically, in tinnitus participants, sustained attention improved with increased anxiety and deteriorated with sleep deprivation, whereas control participants maintained stable performance across these variables. **D to G: Results of the SART. D and E.** Mean reaction times during the SART. The interaction obtained between Group and Hearing loss in the main analysis was not significant in this subgroup (D). The interaction between group and anxiety was marginally significant (p<.10) in this analysis (E) **F.** Principal Component Analysis on thought content during SART task interruptions revealed three primary components: planning-related thoughts (Component 1), task-related thoughts (Component 2), and memory-related thoughts (Component 3). **G**. Between-group analysis of Component 1 revealed significantly more planning-related thoughts in the control group compared to the tinnitus group. Error bars represent the SEM, error ribbons represent the 95% CI.

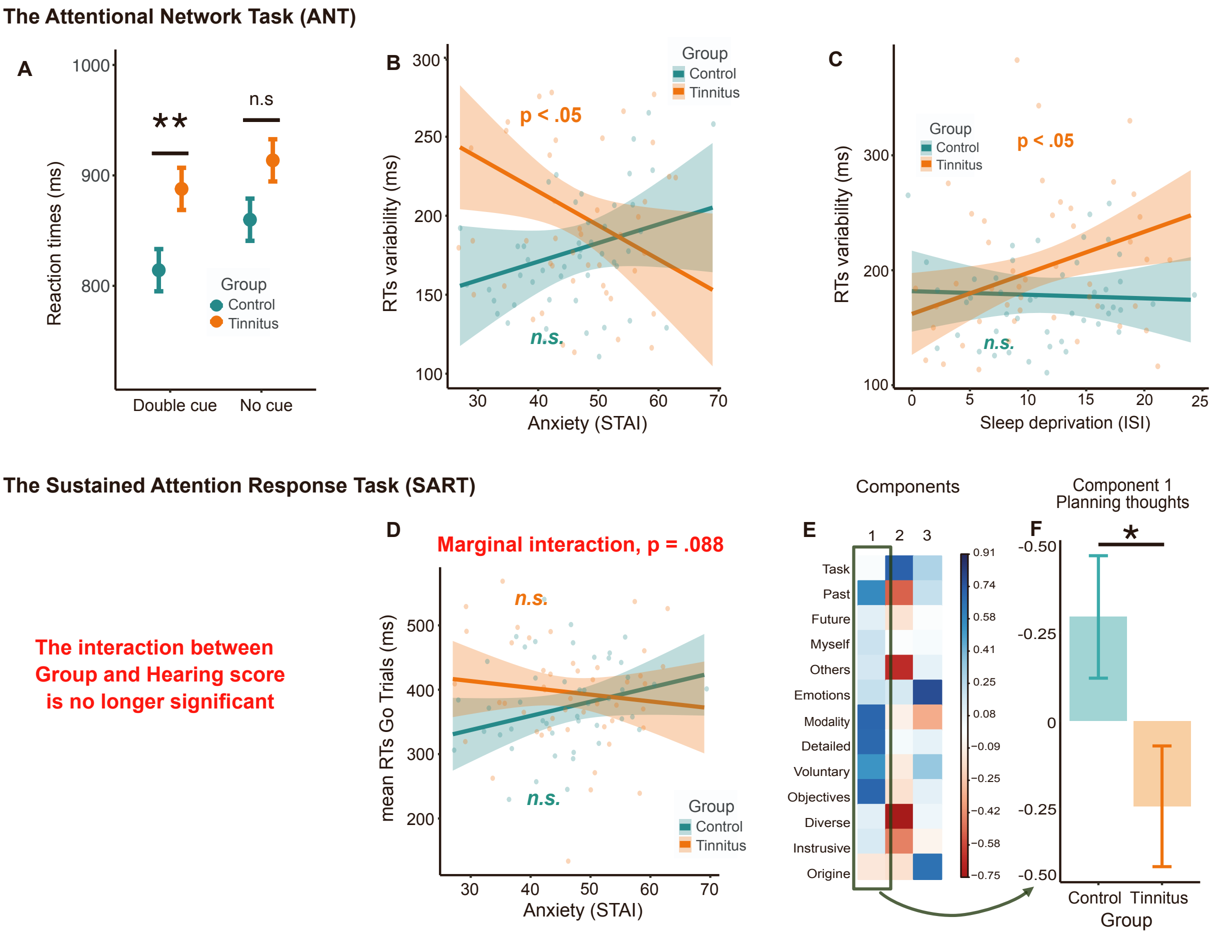

Figure S4: Links between tinnitus severity and sustained attention. We performed correlation between tinnitus characteristics (THI global score, THI functional subscore, and tinnitus duration) and attentional scores (alerting, sustained attention in ANT and in SART). In the ANT, the analysis showed a correlation between THI functional subscore and sustained attention (**A**). In the SART, there were correlations between THI global (**B**), THI functional score (**C**), and sustained attention.

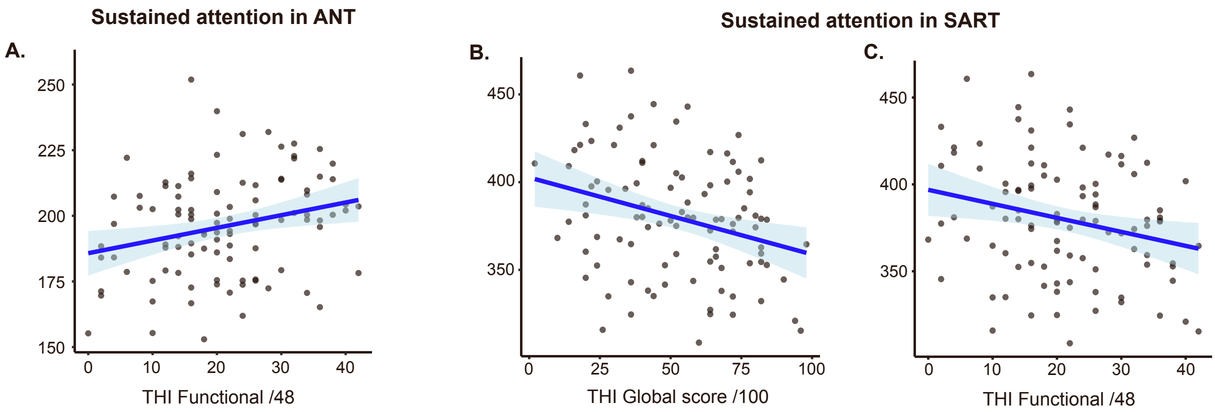

Table S1: Emotional evaluation: matrix of correlation between the difference measures. Correlations with r >.70 are in bold.

	HADS Depression	HADS Anxiety	STAI	PSS4	Khalfa	Sleep disorders
**HADS Depression**		r = 0.582<br>p<.001 ***	**r = 0.720**<br>**p<.001** ***	r = 0.666<br>p<.001 ***	r = 0.439<br>p<.001 ***	r = 0.522<br>p<.001 ***
**HADS Anxiety**			**r = 0.724**<br>**p<.001** ***	r = 0.633<br>p<.001 ***	r = 0.412<br>p<.001 ***	r = 0.534<br>p<.001 ***
**STAI**				**r = 0.722**<br>**p<.001** ***	r = 0.443<br>p<.001 ***	r = 0.503<br>p<.001 ***
**PSS4**					r = 0.368<br>p<.001 ***	r = 0.465<br>p<.001 ***
**Khalfa**						r = 0.294<br>p<.001 ***
**Sleep disorders**						

Table S2: Tinnitus and control group descriptive data. Demographic status, emotional state, sleep disorder and hearing functioning of the two groups of participants.

	**Control**<br>N= 47	**Tinnitus**<br>N= 47	**Test**	**p value**
**Demographic**				
Age	43 ± 12	43 ± 13	Student’s *t*-test	ns
Sex (F/M)	27/20	26/21	Pearson’s chi² test	ns
Laterality (Left/ Ambidextrous/ Right)	7/1/39	6/0/41	Pearson’s chi² test	ns
Years of education	15 ± 3	16 ± 3	Student’s *t*-test	ns
**Emotional state**				
Anxiety (STAI) / 80	46 ± 9	46 ± 10	Student's *t*-test	ns
Anxiety (HADS-A) /21	8 ± 4	9 ± 5	Student's *t*-test	ns
Depression (HADS-D) /21	6 ± 4	6 ± 4	Student's *t*-test	ns
Stress (PSS4) /16	7 ± 4	7 ± 4	Student's *t*-test	ns
Sleep disorder (ISI) /28	11 ± 7	12 ± 7	Student's *t*-test	ns

Data S1. Supplemental Analyses

Section S1: Analysis of the other contents of the Mind Wandering

The analysis of the *first component* (task-related thoughts) indicated an effect of Age (Wald χ^2 = 11.6, p < .001), with more task-related thoughts with aging. There was an effect of the Anxiety (Wald χ^2 = 7.9, p = .005), with less task-related thoughts when anxiety increases.

The analysis of the *third component* (emotional thoughts) indicated an effect of the Level of Education (Wald χ^2 = 7.5, p = .006), with higher education related to more negative thoughts. There was an effect of Anxiety (Wald χ^2 = 18.7, p < .001), with higher anxiety levels related to more negative thoughts.

Section S2: Sensitivity analysis

Participants matching
The groups' (Tinnitus /Control) matching was performed using a propensity-score including demographic measures (Age and Sex) and measures linked to tinnitus comorbidities: hearing threshold (from 125Hz to 16kHz), anxiety scores (STAI and HADS-A), Depression (HADS-D), Stress (PSS4) and Sleep deprivation (ISI). We obtained two groups described in the following table (Table S2). We verified that the participants were well matched in terms of age, sex, emotional state and sleep quality. We also verified the matching in hearing abilities by looking at the pure tone audiometry, but also at the evoked otoacoustic emissions (EOAE) and the mean distortion products otoacoustic emissions (see Figure S2 A to C). All the analysis revealed no group differences.

Statistical analysis
We ran on this subsample similar analysis than in the full sample. Compared to the main analysis in the manuscript, the Hearing score used in this analysis was not the result of the SPLS-DA. Instead, we calculated the mean of the hearing thresholds to account for the variability linked to hearing loss.

Attentional Network task
The analysis of the *Alerting* revealed significant effects of Type of Cue (Wald χ^2 = 57.8, p < .001), of Hearing loss (Wald χ^2 = 4.8, p < .05), of Group (Wald χ^2 = 6.0, p = .03) and a significant interaction between Group x Type of cue (Wald χ^2 = 4.4, p = .04). As seen in Figure S3.A, Tinnitus participants were slower than Control participants after a double cue (planned comparison: p < .01, Bonferroni correction), but there was no group difference in the absence of a cue. This result indicates a lower speeding benefit of an alerting cue in presence of tinnitus.

The analysis of the *Sustained attention* revealed an effect of Group (F(1,81) = 5.2, p < .05) with more variable RTs in the Tinnitus group. Analysis also revealed an interaction between Group x Anxiety (F(1,81) = 6.7, p = .01): the RT variability of participants with tinnitus decreased as anxiety increased (slope different from zero: p =.01), which was not the case for control participants (Figure S3.B). There was also an interaction between Group x Sleep disorder (F(1,81) = 4.1, p = .04): the RTs variability among participants with tinnitus increased with sleep disorders (slope different from zero: p = .03), which was not the case for control participants (Figure S3.C). Globally, participants with tinnitus presented a deficit in sustained attention, modulated by their level of anxiety and sleep, which was not the case for control participants.

Sustained attention to Response task (SART)
Analysis of the *RTs Go trials* evidenced a marginal interaction between Group and Anxiety (F(1,81) = 3.7, p = .088), with RTs decreasing with anxiety in the tinnitus groups and increasing in the control group (slopes are not statistically different from 0) (Figure S3.D). The interaction between Group and Hearing loss was not significant in this analysis (F(1,81) = .45, p = .51).

The answers to the *Mind wandering* probe questions were first analyzed using a PCA, with the extraction of three components (see Figure S3.E). According to the highest loadings associated with each component, we can describe the **first component** as thoughts using words, specific, on solution or objectives, voluntary and on past events. The **second component** was thoughts related to the task, not on events from the past, not on other persons, and not on several subjects, and not intrusive. The **third** was thoughts with an emotional valence, from information in their memory.

The analysis of the *second component* (planning thoughts) indicated an effect of Group (Wald $\chi^2 = 4.5$, $p < .05$), indicating that participants with higher education exhibited more planning-related thoughts, while the control group demonstrated significantly more planning thoughts than the tinnitus group (see Figure S3.F). Conversely, the analysis of the *second component* (task-related thoughts) *and third component* (memory-related thoughts) did not reveal effects of Group.
